# A single dose of African horse sickness virus (AHSV) VP2 based vaccines provides complete clinical protection in a mouse model

**DOI:** 10.1016/j.vaccine.2018.09.065

**Published:** 2018-11-12

**Authors:** Mine Aksular, Eva Calvo-Pinilla, Alejandro Marín-López, Javier Ortego, Adam C. Chambers, Linda A. King, Javier Castillo-Olivares

**Affiliations:** a Department of Biological & Medical Sciences, Oxford Brookes University, Oxford OX3 0BP, UK; b Oxford Expression Technologies Ltd, Gipsy Lane, Oxford OX3 0BP, UK; c The Pirbright Institute, Ash Road, Woking, Surrey GU24 0NB, UK; d CISA-INIA, Valdeolmos, Madrid, Spain; e Department of Veterinary Medicine, University of Cambridge, Madingley Road, CB3 0ES Cambridge, UK

**Keywords:** African horse sickness, Vaccine, MVA, Baculovirus, AHSV-VP2

## Abstract

African horse sickness is a severe, often fatal, arboviral disease of equids. The control of African horse sickness virus (AHSV) in endemic countries is based currently on the use of live attenuated vaccines despite some biosafety concerns derived from its biological properties. Thus, experimental vaccination platforms have been developed over the years in order to avoid the biosafety concerns associated with the use of attenuated vaccines.

Various studies showed that baculovirus-expressed AHSV-VP2 or modified Vaccinia Ankara virus expressing AHSV-VP2 (MVA-VP2) induced virus neutralising antibodies and protective immunity in small animals and horses.

AHSV is an antigenically diverse pathogen and immunity against AHS is serotype-specific. Therefore, AHS vaccines for use in endemic countries need to induce an immune response capable of protecting against all existing serotypes. For this reason, current live attenuated vaccines are administered as polyvalent preparations comprising combinations of AHSV attenuated strains of different serotypes.

Previous studies have shown that it is possible to induce cross-reactive virus neutralising antibodies against different serotypes of AHSV by using polyvalent vaccines comprising combinations of either different serotype-specific VP2 proteins, or MVA-VP2 viruses. However, these strategies could be difficult to implement if induction of protective immunity is highly dependent on using a two-dose vaccination regime for each serotype the vaccine intends to protect against.

In our study, we have tested the protective capacity of MVA-VP2 and baculovirus-expressed VP2 vaccines when a single dose was used. Groups of interferon alpha receptor knock-out mice were inoculated with either MVA-VP2 or baculovirus-expressed VP2 vaccines using one dose or the standard two-dose vaccination regime. After vaccination, all four vaccinated groups were challenged with AHSV and clinical responses, lethality and viraemia compared between the groups. Our results show that complete clinical protection was achieved after a single vaccination with either MVA-VP2 or baculovirus sub-unit VP2 vaccines.

## Highlights

• Baculovirus-expressed AHS-VP2 and MVA-VP2 vaccines were evaluated in mice.
• Clinical protection was complete in mice receiving one or two doses of MVA-VP2.
• Clinical protection complete after two doses of baculovirus-expressed VP2.
• Significant reduction of viraemia in all vaccinated groups.
• Significant levels of immunity were achieved with one dose of either vaccine.

## 1 Introduction

African horse sickness (AHS) is a viral disease of *Equidae* transmitted by haematophagus insects of the genus *Culicoides*. The disease, which in horses is extremely severe and often fatal, is caused by African horse sickness virus (AHSV), a member of the genus *Orbivirus*, family *Reoviridae*, closely related to bluetongue virus (BTV). AHSV is a spherical virion of 55–70 nm in diameter, is not enveloped and its icosahedral capsid is formed by three concentric protein layers. The capsid encloses the 10 double-stranded RNA segments of the genome and the virus replication complex formed by VP1, VP4 and VP6, which are encoded by genome segments 1, 4 and 9 respectively [1], [2]. The inner capsid protein VP3, encoded by segment 3, forms the icosahedral scaffold for the outer core VP7 protein. The outer capsid layer is formed by two major structural proteins, VP2 and VP5 (encoded by segments 2 and 6 respectively), involved in cell attachment and entry. VP2 is the most variable antigen of AHSV, determines serotype formation [3] and contains most of the virus neutralising antibody (VNAb) epitopes identified so far [3], [4], [5].

Control of AHS relies on a range of measures of which vaccination plays a critical role. Live attenuated AHS vaccines have been in use in Africa for almost 100 years and are the only ones currently licensed [6], [7], [8]. This type of vaccines is not regarded as entirely safe because of the capacity of vaccine viruses to revert to virulence, their ability to exchange genome segments with field strains, and for their teratogenicity. Recent studies indicate that outbreaks of AHS occurring in the Western Cape Province of South Africa between 2004 and 2014 resulted from re-assortment events involving vaccine strains of serotype 1 [9]. The drawbacks of live attenuated vaccines together with their inability to discriminate vaccinated from infected animals in vaccinated horse populations make their use highly debatable, especially in non-endemic countries.

Over the last 30 years much attention has been given to the development of safer alternative AHS vaccines and a number of different approaches have been explored. Some of these approaches exploit the power of reverse genetics [10] to generate gene defective (yet biologically active) AHSV with impaired replication capacity [11], [12], [13], [14], [15].

Other strategies are based on VP2 and/or VP5, which bear virus neutralizing epitopes, and include baculovirus-expressed VP2 sub-unit vaccines [16], [17], [18] or recombinant viral vector vaccines (Canarypox or Modified Vaccinia Ankara [MVA]) expressing these antigens [19], [20], [21], [22], [23], [24], [25]. In the case of baculovirus VP2 vaccines, the protein is produced *in vitro* and then administered as a cell lysate with an adjuvant. Canarypox VP2/VP5 viruses are formulated with a Carbomer adjuvant and immunogenicity is expected to depend on expression of VP2/VP5 from within cells of the vaccinated host after inoculation. The immunogenicity of experimental MVA-VP2 vaccines, rely on the expression of VP2 from host cells after vaccination and also on presence of pre-formed VP2 in the vaccine inoculum [19]. All these vaccines have been shown to be protective and rely on the efficient induction of virus neutralizing antibodies (VNAb), which typically occur after a primary course of two vaccinations. However, it is not known what levels of immunity would be obtained after a single dose. The availability of this information would be important for the development of polyvalent vaccines for AHS based on these strategies as it would enable to reduce the costs of production and the number of vaccine doses to be given.

Indeed, AHS immunity is serotype specific and vaccines for AHSV need to induce protective immunity across all nine serotypes, especially if they are to be used in endemic countries. For this reason, live attenuated vaccines are formulated as polyvalent vaccines comprising combinations of different strains representing different serotypes [26]. Thus, a typical vaccination course comprises two inoculations: one dose containing serotypes 1, 3 and 4, followed by a second dose containing serotypes 2, 6, 7 and 8 administered one month later. Generating polyvalent AHSV vaccines using recombinant baculovirus-expressed VP2, MVA-VP2 or Canarypox VP2/VP5 would require combining single serotype-specific constructs and some studies indicate that this is possible [16], [20]. Such polyvalent vaccination strategies would be easier to implement if protection could be achieved after one vaccination dose.

In this study, we examined, in a vaccination and challenge experiment in a mouse model, the levels of protection conferred by MVA-VP2 and baculovirus-expressed VP2 vaccines upon a single inoculation.

## 2 Materials and methods

### 2.1 Baculovirus expressed VP2 vaccines

#### 2.1.1 Cells

Insect cell lines Sf9 and Sf21 from *Spodoptera frugiperda* and TnHi5 from *Trichoplusia ni,* were cultured at 28 °C. Sf9 and TnHi5 cells were maintained in ESF 921 serum-free medium (Expression Systems) and Sf21 cells were maintained in TC100 (Gibco) medium supplemented with 10% (v/v) foetal bovine serum (FBS) [27].

#### 2.1.2 Preparation of recombinant baculovirus expressing AHSV4 VP2 protein

Nucleotide sequences encoding AHSV4 VP2 were PCR-amplified from the template vector pSC11-AHSV-4-VP2 [24] using gene-specific primers. A polyhistidine tag (6×His) coding sequence was added to the 5′ terminus of the VP2 sequence during the PCR amplification. The PCR product, VP2^HIS/N^, was sub-cloned into the pGEM-T Easy vector prior to cloning into the pOET-1 baculovirus transfer vector (Oxford Expression Technologies Ltd [OET]) using *Not*I restriction sites to generate the recombinant vector pOET1_VP2^HIS/N^.

Recombinant baculovirus expressing his-tagged AHSV4-VP2 was generated using the *flash*BAC™ system following the supplier’s protocol (OET). Briefly, Sf9 cells (0.7 × 10^6^ cells/dish) were co-transfected with the *flash*BACULTRA virus DNA (100 ng) and the pOET1_VP2^HIS/N^ transfer vector (500 ng) using a liposome-mediated transfection method (Lipofectin™, Invitrogen). Following 5 days of incubation at 28 °C, culture medium containing the recombinant virus, AcVP2^HIS/N^, was harvested (P0 virus seed stock). Subsequently, a 50 ml P1 virus stock was amplified in Sf9 cells maintained in shaker cultures (2 × 10^6^ cells/ml) during 5-day incubation at 28 °C. Virus stocks were titrated using a QPCR-based titration system following the supplier’s protocol (baculoQUANT™, OET) [28]. Subsequent titrations were carried out using the standard plaque assay system in Sf21 insect cells as described previously [27].

#### 2.1.3 Preparation of purified VP2 protein

TnHi5 insect cells seeded in large tissue culture dishes (1.7 × 10^7^ cells/dish, 38 dishes in total) were infected with AcVP2^HIS/N^ using a multiplicity of infection (moi) of 5. After 72 h incubation at 28 °C, the infected cells were harvested by centrifugation at 4000 rpm for 15 min at 4 °C. The cell pellets were resuspended in lysis buffer (10 mM Tris-HCL, pH 8.0, 0.15 M NaCl, 1 mM EDTA and 1% (v/v) NP-40 supplemented with Protease inhibitor cocktail (Calbiochem)) and incubated on ice for 1–2 h. Following cell lysis, the medium was clarified by centrifuging at 20,000 rpm for 20 min at 4 °C. 0.15 M NaCl and 10 mM imidazole were added to the clarified medium, which was then incubated with nickel-coated sepharose beads (GE Healthcare) on a rotating platform for 2 h at 4 °C. After the incubation, the beads were transferred onto Proteus 1-step batch midi spin columns (Generon) and washed three times using Buffer A (20 mM Tris-HCl, pH 8.0, 0.3 M NaCl, 10 mM imidazole). His-tagged proteins were then eluted from the beads with Buffer B (300 mM imidazole, 20 mM Tris-HCl, pH 8.0 and 0.3 M NaCl) and stored at 4 °C until used.

Samples from the purification fractions were mixed with 1/5th volume of 6X Laemmli SDS-PAGE loading buffer (250 mM Tris-HCl pH 6.8, 10% SDS, 50% (v/v) glycerol, 0.05–0.5% bromophenol blue and 25% (v/v) β-mercaptoethanol) and analysed with SDS-PAGE followed by western blotting and Coomassie staining. Total protein concentration of the eluate samples was estimated with BCA protein assay using the standard micro-scale assay format following the supplier’s instructions (Novagen) and VP2 protein concentration was determined by band densitometry from the Coomassie stained gels.

### 2.2 MVA-VP2 vaccines

Vero cells (ATCC, Cat. No. CCL-81) and Chicken embryo fibroblast (DF-1) (ATCC, Cat. No. CRL-12203) were grown in high glucose Dulbecco’s modified Eagle’s medium (DMEM) supplemented with 2 mM glutamine, penicillin (100 units/ml), streptomycin (100 µg/ml) and 10% foetal calf serum (FCS). MVA-VP2, expressing AHSV-4 VP2, was previously described [19], [22], [23], [24], [29]. The MVA-VP2 vaccine virus was bulked up for this study in DF-1 cells and subsequently used as a cell lysate. Virus stocks were generated by infection of sub confluent cells using a multiplicity of infection (MOI) of 0.1. When a total cytopathic effect (CPE) was visible, the cells and supernatants were harvested and centrifuged. The virus was released from the cells by three freeze/thaw cycles, sonication and then titrated by plaque assay.

### 2.3 Western blot

Immunoblotting was performed as described previously [24]. Samples were mixed 1:5 with 5× Laemmli sample buffer and 10 µL were loaded in each well of polyacrylamide gels. Three immunogenic AHSV-4 VP2-derived KLH-conjugated peptides (NH_2_-KKKEEGEDDTARQEIRKAWC-COOH; NH_2_-NKGKWKEHIKEVTEKLKKA-COOH; NH_2_-DMNEKQKPYFEFEYDDFKPC-COOH) were selected to obtain a VP2-specific rabbit polyclonal antibody from a commercial source (GenScript). This antibody was used at a 1:1000 dilution. As a secondary antibody, a horseraddish peroxidase-labelled goat anti-rabbit IgG (Sigma) was used at a dilution of 1:50,000. Alternatively, detection of VP2 antigen by Western blotting was performed with AHSV-4 VP2-specific monoclonal antibodies (MAb) 8BG9 and 8DB11 (INGENASA, Madrid, Spain), used at a 1:500 dilution, and a horseraddish peroxidase labelled goat anti-mouse IgG (Jackson Immuno Research) as a secondary antibody at a 1:50000 dilution.

### 2.4 Mice

Seven-week-old, female, Type I interferon receptor KO A129 IFNAR (−/−) mice were purchased from B&K Universal. The animals were rested for about a week before the experiments were performed in the animal facilities of the Centro de Investigación en Sanidad Animal (INIA-CISA). All protocols for animal use were approved by the Ethical Committee of the Centre for Animal Health Research (CISA-INIA) (Permit number: PROEX 039/15) in strict accordance with the Spanish National Royal Decree (RD1201/2005), EU guidelines 2010/63/UE about protection of animals used for experimentation and other scientific purposes, and the Spanish Animal Welfare Act 32/2007.

### 2.5 Vaccination of animals.

Five groups of IFNAR –\– mice (n = 6) were used in the study (Table 1). Groups 1 and 2 were vaccinated with one or two doses of MVA-VP2 at 10^7^ pfu/mouse respectively. Groups 3 and 4 were vaccinated with one or two doses of Alum adjuvanted VP2^HIS/N^ at 10 µg per mouse. Baculovirus-expressed VP2 in saline was added to Alum at a 1:1 ratio and administered to mice. Group 5 was not vaccinated and used as control.

**Table 1:** Vaccination groups and dosage.

Group	Vaccine	Dose	Number of doses	Days of vaccination
1	MVA-VP2	10^7^ pfu/ml	1	Day 0
2	MVA-VP2	10^7^ pfu/ml	2	Days 0 and 21
3	VP2	10 µg	1	Day 0
4	VP2	10 µg	2	Days 0 and 21
5	PBS		2	Days 0 and 21

### 2.6 Plaque reduction neutralization test (PRNT)

Serial dilutions of mouse sera were incubated with 100 pfu of AHSV-4 for 1 h at 37 °C. Then, samples were inoculated into one well each of 12-well plates containing confluent monolayers of Vero cells. Following incubation for 1 h at 37 °C in 5% CO_2_ an agar overlay (DMEM, 10% FBS, 0.4% agar) was added and plates incubated further for 5 days at 37 °C in 5% CO_2_. Plaques were visualized with a counter-stain solution (2% crystal violet, 10% formaldehyde, PBS). PRNT titre was calculated as the reciprocal (log_10_) of the highest dilution of serum that neutralised 50% of the control virus input.

### 2.7 Detection of antibodies against VP2 by ELISA

MaxiSorp plates (Nunc, USA) were coated with VP2 purified baculovirus expressed proteins (164 ng per well) and incubated overnight at 4 °C. Plates were saturated with blocking buffer (PBS-0.05% Tween 20 and 5% skim milk). The animal sera diluted in blocking buffer were added and incubated for 1 h at 37 °C. After three washes in PBS-0.05% Tween 20, plates were incubated for 1 h at 37 °C with an anti-mouse-HRP secondary antibody (Biorad, USA) at a 1:2000 dilution in blocking buffer. Finally, after three washes in PBS-0.05% Tween 20, the reaction was developed with substrate solution tetramethylbencidine liquidsupersensitive (TMB) (Sigma) and stopped by adding 50 ml of 3 N H_2_SO_4_. Results were expressed as optical densities (ODs) measured at 450 nm. Background OD values were obtained from wells containing blocking buffer without serum samples. This background OD was subtracted from the OD measurements of the sample wells.

### 2.8 Challenge with AHSV

The AHSV-4 virus stock used for challenge and for virus neutralisation tests has been previously described [23]. This was derived from a Spanish isolate of AHSV (Madrid-87), which was passaged twice in mouse brain and three times in BHK-21 cells before stocks for this study were prepared. These were grown in Vero cells and used to challenge the mice by sub-cutaneous injection of 10^6^ pfu/mouse 5 weeks after all vaccinated mice received the first vaccination. Animals were monitored twice a day after challenge and clinical signs recorded. Mice were sampled at regular intervals for analysis of virus load in blood.

### 2.9 Clinical signs and viraemia

Clinical scoring was performed as described previously [29]. Mice were humanely euthanized when they showed severe clinical signs (weight loss, dehydration, frequent hunching, severe conjunctivitis or any other condition that prevented food or water intake). Whole-EDTA-blood samples were collected from the submandibular vein at different days post infection to analyse viraemia. Blood cells were lysed with water before performing plaque assays on Vero cells as described previously [23].

### 2.10 Statistical analysis

Differences in antibody levels between groups inoculated with various AHSV-VP2 vaccines (groups 1 to 4) were determined using the Sidak-Bonferroni method. Clinical scores in different groups of mice were compared by Kruskal-Wallis test. Differences between groups were then explored using Wilcoxon rank-sum tests. Titres of viraemia were compared using a Kruskal-Wallis test, followed by Mann-Whitney non-parametric tests to identify differences between groups at 4 and 7 dpi. Survival data were analysed using a log rank test with mice grouped by immunisation strategy. A significance level of P = 0.05 was used in all analyses.

## 3 Results

### 3.1 Production and optimizing the yield of baculovirus expressed AHSV4-VP2

The first stage for preparing VP2 based vaccines was the production and purification of the VP2 protein in the baculovirus system. Preliminary experiments showed that expression levels of AcVP2^HIS/N^ in Sf9 cells were variable and often the protein was not detectable by SDS-PAGE and Coomassie blue staining. Therefore, we also explored the potential of TnHi5 cells to support the production of VP2 following infection with AcVP2^HIS/N^. Thus, Sf9 and TnHi5 cells were infected with AcVP2^HIS/N^ using a multiplicity of infection (MOI) of 5 pfu/cell and the expression levels of AHSV VP2 was analyzed after harvesting the cells at 72 h post-infection.

Recombinant VP2 was clearly detected from crude AcVP2^HIS/N^ infected TnHi5 cell lysates using both anti-Histidine and anti-VP2 specific antibodies (Fig. 1A). In contrast, VP2 could not be detected, with either antibody, in crude AcVP2^HIS/N^ infected Sf9 cell lysates in the immuno-blots.

**Fig. 1A:**
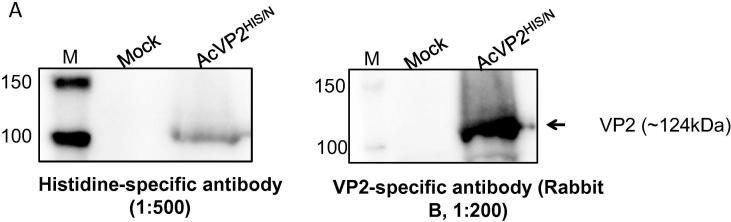
Analysis of VP2^HIS/N^ production in TnHi5 insect cells. TnHi5 cells (0.5 × 10^6^ cells/dish) were infected with AcVP2^HIS/N^ using 5 moi. The cells were harvested at 72hpi by centrifugation at 13000 rpm for 5 min and VP2 production was analysed by SDS-PAGE, followed by western blotting using histidine- and VP2-specific antibodies.

Purification of VP2, expressed from AcVP2^HIS/N^ infected Sf9 or TniHi5 cells, was performed by affinity chromatography. Although affinity-purified VP2 could be detected by Western blotting in both Sf9 and in TnHi5 cells, expression levels in Sf9 were not sufficiently high for detection by Coomassie blue staining in SDS-PAGE gels (data not shown). In contrast, VP2 was expressed abundantly in TnHi5 cells as evidenced by the detection of protein bands of approximately 124 kDa in several eluate fractions by Western blotting and Coomassie blue staining (Fig. 1B). Consequently, TnHi5 cells were used for the preparation of the VP2 vaccine stocks for this vaccination study.

**Fig. 1B:**
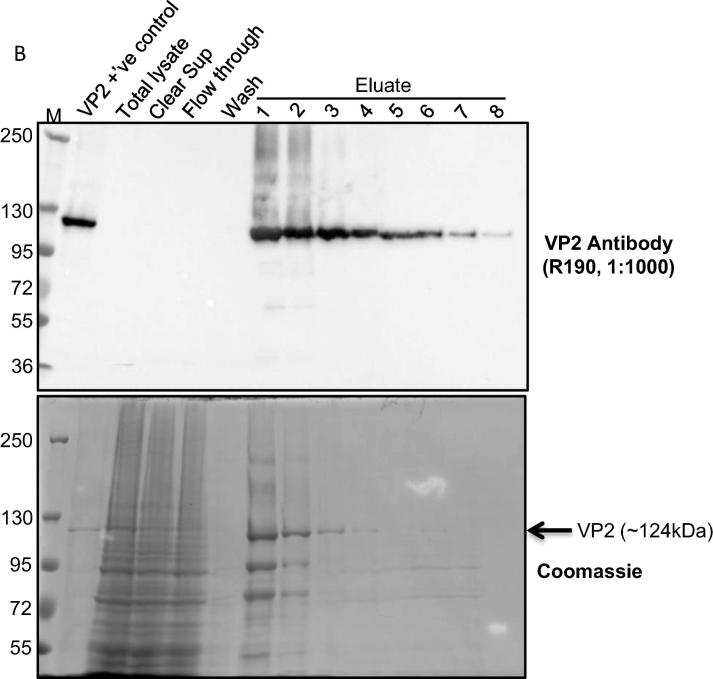
Analysis of VP2^HIS/N^ purification. TnHi5 cells (1.7 × 10^7^ cells/dish) were infected with AcVP2^HIS/N^ using 5 moi. The cells were harvested at 72 hpi by centrifugation at 4000 rpm, 15 min at 4 °C and VP2^HIS/N^ was purified using nickel-coated sepharose beads. Purified protein was collected in 8x1ml eluate fractions. Purification fractions were analysed by SDS-PAGE, followed by western blotting (top) and Coomassie Brilliant blue R-250 staining (bottom). Previously purified VP2 was used as a positive control in the protein analysis. Only Eluate 1 was used in the vaccine preparation. (For interpretation of the references to colour in this figure legend, the reader is referred to the web version of this article.)

The total protein concentration, measured by BCA protein assay, in individual eluate samples collected from AcVP2^HIS/N^ infected TnHi5 cells (6.5 × 10^8^ cells in 950 ml) was estimated to be 318 µg from eluate 1 and 179 µg from eluate 2. Only the first eluate was used for vaccine preparation.

The antigenicity of purified VP2 was further confirmed by immunoblotting with VP2-specific MAb in addition to VP2-specific rabbit polyclonal antiserum. Both MAbs reacted with the purified protein (Fig. 1C).

**Fig. 1C:**
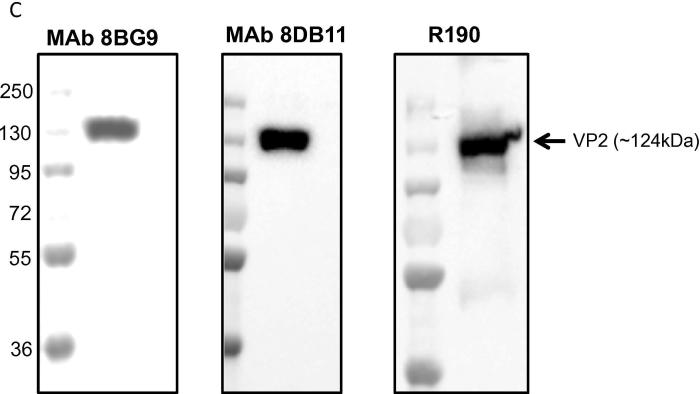
Antigenic reactivity of purified VP2^HIS/N^ to AHSV4-specific monoclonal antibodies. Purified VP2^HIS/N^ was analysed by SDS-PAGE followed by western blotting using two monoclonal (8BG9 and 8DB11) and a polyclonal (R190) AHSV4-VP2 specific-antibodies.

### 3.2 Antibody responses following vaccination

All vaccinated mice developed VNAb against AHSV following vaccination (Table 2). One animal of group 2 died at day 15 without showing clinical signs the previous days; therefore it was considered that this death was not related to the vaccination. Pools of sera from each group were analysed in duplicate. The serum VNAb titres before challenge (day 35) of groups 2 and 4, which received two vaccine doses, were higher (mean PRNT 2.34 and 1.87 respectively) than those of groups 1 and 3 (mean PRNT 1.69 and 1.39 respectively), which received just one vaccination dose. There were statistically significant differences in VNAb titres among groups (P < 0.05).

**Table 2:** Results of PRNT test and ELISA of serum samples collected from mice groups 1–4 on day 35 post-vaccination.

Group	Mean Log_10_ PRNT_50_ ± SD	Mean OD ELISA VP2 ± SD
1	1.695 ± 0.049	0.99 ± 0.27
2	2.34 ± 0.071	1.70 ± 0.18
3	1.365 ± 0.177	0.42 ± 0.27
4	1.869 ± 0.015	1.65 ± 0.7

The presence in serum of specific antibodies to VP2 was also analysed before challenge by indirect

ELISA using individual samples. Responses measured by ELISA indicated that, as expected, the OD values of the negative control were very low (mean OD 0.044). Results of the vaccinated groups were consistent with VNAb data, indicating that higher antibody responses were obtained for groups 2 and 4 (mean OD 1.7 and 1.65 respectively) than for groups 1 and 3 (mean OD 0.99 and 0.42 respectively). In this case, there were significant differences (P = 0.005) between groups 1 (MVA-VP2) and group 3 (baculovirus expressed VP2 vaccine) when one dose was used. In contrast, there was not a statistical difference between groups 2 and 4, indicating that both vaccines were effective at inducing VP2 antibodies in these vaccine groups. However, MVA-VP2 vaccines induced higher titres than baculovirus-expressed VP2 vaccines. As expected, there were significant differences between animals vaccinated with one or two doses, i.e. between groups 1 and 2 (P = 0.004); and between 3 and 4 (P = 0.004).

### 3.3 Challenge

**Fig. 2:**
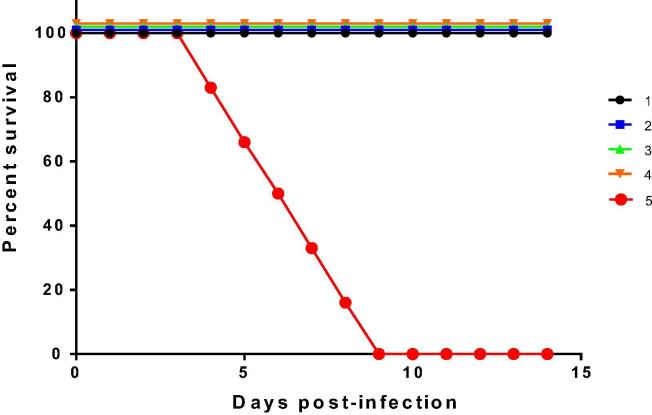
Survival rates of immunized and non-immunized IFNAR (–/–) mice after inoculation with AHSV-4. Groups of mice 1 to 4 (n = 6) were immunized with MVA-VP2 or baculovirus VP2 protein after a single or double vaccination. Non-immunized group 5 was inoculated with PBS and used as a control. At day 35 all mice were subcutaneously inoculated with 10^6^ PFUs of AHSV-4. The mice were observed every 24 h for 14 days.

Three weeks following the last vaccination all mice, including the negative control group, were challenged with AHSV-4 and protective efficacy was assessed. None of the non-vaccinated controls survive the challenge. In contrast, all vaccinated mice survived the infection. Clinical signs were significantly reduced in comparison with the unvaccinated controls (group 5). Clinical signs in group 5 started on day 3 post-infection and reached the humane end-point between days 4 and 9 post-challenge (Fig. 2). These signs included dehydration, ocular discharges, periorbital swelling and hunching. In contrast, mice from all other groups survived the challenge and developed no clinical signs (except one mouse in group 3). Survival was significantly (P < 0.0001) lower in unvaccinated mice compared to all other groups.

**Fig. 3:**
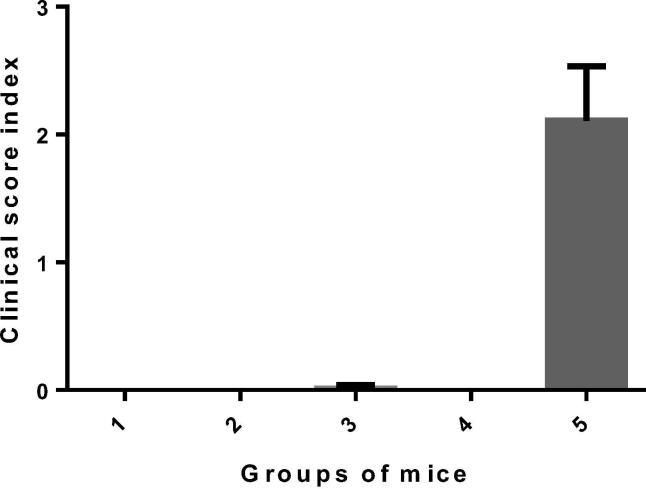
Clinical score index of groups of mice after infection with AHSV-4. A clinical scoring system was used whereby each animal received a clinical score value per day. The value used for analysis was the individual mean clinical score for the study period (sum of daily scores of each animal/number of days). Standard deviations are shown as error bars.

Clinical scores were negative for mice receiving one or two doses of MVA-VP2 and for mice receiving two doses of baculovirus expressed VP2 vaccine (group 4). Clinical signs were highly reduced in animals vaccinated with one dose of the baculovirus expressed VP2 as only one mouse of group 3 displayed mild clinical signs (Fig. 3). The average clinical score index for group 5 was 2.11 ± 0.42, which was statistically higher than the clinical scores recorded for the other vaccinated groups (P = 0.03), as expected. The clinical protection afforded by a single dose of either vaccine was evident and there were no statistical differences between the levels of protection observed for vaccinated groups despite some subtle clinical signs were observed in group 3.

**Fig. 4:**
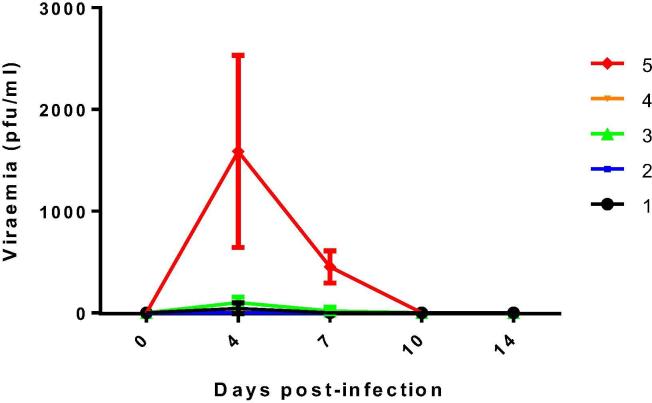
Titres of AHSV-4 recovered in blood of immunized and non-immunized IFNAR (–/–) mice after challenge. Analysis of infectious virus was performed at 0, 4, 7, 10 and 14 dpi by plaque assay. Each point represents the mean value of the viral titre of six animals, and standard deviations are shown as error bars.

A statistically significant reduction of viraemia (P < 0.05) was observed in all vaccine groups relative to the unvaccinated controls. Thus, AHSV concentration in blood was below the threshold of detection of the assay in animals from Group 2 during the whole study period (Fig. 4). Some individuals of the other vaccinated groups developed low levels of viraemia in some animals: three in group 1; one in group 4; and all animals in group 3. However, viraemia was cleared by day 7 p.i. in groups 1 and 4, and by day 10 p.i. in group 3. The levels of viraemia on days 4 and 7 p.i. were significantly lower (P = 0.002 and P = 0.01 respectively) in vaccinated groups relative to the unvaccinated group. High viral loads were detected in unvaccinated mice, reaching maximum values between days 4 and 7post-challenge. The average viral titre in group 5 was 1587.5 pfu /ml at day 4 p.i. and 450 pfu/ml at day 7 p.i.

All these results indicated that vaccination with MVA-VP2 or baculovirus-expressed VP2 induced protective immunity against AHSV, even after using a single vaccine dose. This protection was characterised by a complete prevention of lethality of AHSV infection in all vaccinated groups, an absence of viraemia in group 2 and a significant reduction of clinical signs and viraemia in groups 1, 3 and 4 relative to the control group. The level of protection observed in the different groups of vaccinated mice seems to correlate with antibody response against AHSV (Table 3). Viraemia, viral load, lethality and clinical scores are directly proportional to the VNAb pre-challenge.

**Table 3:** Correlation VNAb with protection.

Level of protection	Group	Log_10_ PRNT_50_*	OD ELISA VP2*	Clinical protection	Viraemia	Viral load (pfu/ml)*	Survival
High	2	2.34	1.70	5/5	0/0	0	5/5
High/medium	4	1.87	1.65	5/5	1/5	8.33	5/5
High/medium	1	1.69	0.99	5/5	3/5	41.66	5/5
Medium	3	1.36	0.42	4/5	5/5	100	5/5
Low	5	< 0.69	0.044	0/5	5/5	1587.5	0/5

* Mean values of the group.

## 4 Discussion

Previous studies in the mouse model with experimental AHSV MVA-VP2 vaccines indicated that VNAb are an important effector mechanism of immunity against AHSV [22], [29]. Immunogenicity of live attenuated, inactivated and DISC and DISA vaccines is normally studied in relation to the levels of VNAb induced in the vaccinated animals, although studies in mice and horses indicate that cell-mediated immunity seems to also play a role in protection against AHSV [29], [30], [31]. To date, no published studies have specifically established the minimum VNAb titre in serum required to provide immunity against AHSV in horses. However, the vaccine manufacturer of the attenuated vaccine relies on the induction of a titre of 1/16 as minimum requirement for protection in horses [32] and other data suggest that a titre of 1/64 would confer solid immunity against AHSV with prevention of clinical signs and viraemia also in horses (Baltus Erasmus, personal communication). High levels of VNAb titres are seldom achieved by a single immunization with any type of AHSV vaccine. This is the reason why protection efficacy experiments are rarely performed after a single vaccination, when VNAb titres are still low.

In this study, we have evaluated, in the IFNAR –/– mouse model, the protective capacity against virulent AHSV challenge, of recombinant MVA-VP2 and baculovirus-expressed VP2 vaccines following a single vaccine dose. For this, we have vaccinated groups of 6 mice with either MVA-VP2 or baculovirus-expressed VP2 adjuvanted vaccines using either one or two doses and challenged the vaccinates with AHSV. Clinical signs, survival and viraemia were compared between the vaccinates and a negative control group of mice which was challenged at the same time of the vaccinates. We showed that protection against lethality, clinical signs and viraemia in all vaccine groups was very high and statistically significant respect to the control group. As expected, individuals vaccinated with two doses were best protected, but even those receiving just one dose showed high levels of protection in the face of relatively low serum VNAb titres at the time of challenge.

The results above might suggest that cell mediated immunity contributed to the protection levels observed in animals vaccinated with one dose. Indeed, we showed previously that MVA-VP2 vaccines induce cellular immunity and that transferred splenocytes from vaccinated mice to naïve recipients conferred certain degree of immunity in the latter [22]. However, it is also possible that priming the immune system, through MVA-VP2 or baculovirus-expressed VP2 immunisation, enabled a strong anamnestic VNAb response upon a second encounter with the VP2 antigen when the vaccinated mice were challenged with AHSV and that this was sufficient to prevent clinical signs and lethality in the vaccinates. Protective efficacy of one-dose vaccination has also been proven by other studies that used different vaccine platforms against various disease targets such as porcine circoviral disease, PCVD, [33], influenza [33], [34], [35], classical swine fever, CSF, [36], Ebola and Marburg [37].

Achieving protective immunity by a single vaccination could be a major step forward in the development of viral vector vaccines for an antigenically plural pathogen such as AHSV, especially if the vaccine is for use in endemic countries where protection against several serotypes is required. Recently, Kanai and co-workers demonstrated the potential to generate polyvalent AHSV vaccines using baculovirus-expressed AHSV-VP2 in a serological study in mice [16]. Similarly, previous vaccination studies in ponies by Manning and co-workers, demonstrated that recombinant MVA-VP2 vaccines can potentially be used in polyvalent formulations [20]. Indeed, the latter study showed that simultaneous administration of two recombinant MVA-VP2 vaccines, each expressing an AHSV VP2 protein from a different serotype (one from serotype 4 and another one from serotype 9) followed by a booster vaccination 5 months later with a recombinant MVA-VP2 vaccine specific for another serotype (AHSV-5), resulted in the induction of antibodies that neutralized the infectivity of 5 AHSV serotypes. However, the induction of high VNAb was achieved after 2 vaccine doses. This requirement would have implications in the design of the vaccination regime as well as vaccine production costs. Therefore, the possibility of achieving protection after a single dose of MVA-VP2 or baculovirus-expressed VP2 vaccination could reduce the number of doses to be given, facilitate the design of the vaccination regime and reduce costs of vaccine manufacturing.

In our study, the protection conferred by the baculovirus-expressed VP2 vaccine was comparable to that of MVA-VP2 vaccines, in particular when two doses of the baculovirus expressed VP2 vaccine were used. This finding is not surprising as other researchers previously showed the protective capacity of sub-unit vaccines based on VP2. However, previous studies with baculovirus-expressed AHSV-VP2 vaccines used *Spodoptera frugiperda* cells, mainly Sf9, as the target host for production of the vaccine [16], [17], [38], [39], [40]. Some of these studies showed that the production yields of VP2 from AHSV4 [38] and AHSV5 [39] using Sf9 cells were low and most of the protein was insoluble. Further analysis carried out in guinea pigs [39] and horses [17] showed that the generation of VNAb is dependent on the presence of soluble VP2. Production of VP2 in Sf9 cells was also proven to be difficult in our study. However, the use of another insect cell line, *Trichoplusia ni*, (TnHi5) was shown to increase the production yields of VP2 with recombinant baculoviruses. The differences in protein expression profiles between these two cell lines have also been observed in several other studies, where TnHi5 cells were favoured for the expression of recombinant proteins and virus-like-particles [41], [42]. All previous baculovirus-based AHSV-VP2 vaccines were formulated from crude cell lysates or soluble protein extracts [16], [17], [38], [39], [40]. The presence of molecules other than VP2 in the vaccine would make it difficult formulating this type of vaccines and assess their potency *in vitro*. In our study, the baculovirus-expressed VP2 was eluted easily from the affinity columns using imidazole, without using denaturing agents such as Urea or Guanidine Hydrochloride. Although we have not assessed specifically what proportion of the protein was insoluble, our data indicate that most of the protein was indeed soluble. The affinity purification process improves purity of the vaccine preparation reducing the presence of other molecules in the vaccine’s inoculum. To our knowledge, this is the first study to show the protective efficacy of histidine-tagged affinity-purified VP2 in a vaccination-challenge experiment. It is possible that this technical finding could facilitate the production of this type of vaccines in an industrial setting.

Overall, the preliminary results from our study show the potential of generating single-dose AHSV vaccines using baculovirus and MVA platforms. These findings warrant further investigations directed at testing the protective efficacy of polyvalent vaccines based on baculovirus-expressed VP2 and MVA-VP2.
